# The Kalash Genetic Isolate: Ancient Divergence, Drift, and Selection

**DOI:** 10.1016/j.ajhg.2015.03.012

**Published:** 2015-05-07

**Authors:** Qasim Ayub, Massimo Mezzavilla, Luca Pagani, Marc Haber, Aisha Mohyuddin, Shagufta Khaliq, Syed Qasim Mehdi, Chris Tyler-Smith

**Affiliations:** 1 Wellcome Trust Sanger Institute, Wellcome Trust Genome Campus, Hinxton, Cambridgeshire CB10 1SA, UK; 2 Institute for Maternal and Child Health, IRCCS Burlo Garofolo, University of Trieste, 34137 Trieste, Italy; 3 Division of Biological Anthropology, University of Cambridge, Cambridge CB2 1QH, UK; 4 Section of Biochemistry, Shifa College of Medicine, Shifa Tameer-e-Millat University, Sector H-8/4, Islamabad 44000, Pakistan; 5 Department of Human Genetics & Molecular Biology, University of Health Sciences, Lahore 54000, Pakistan; 6 Centre for Human Genetics and Molecular Medicine, Sindh Institute of Urology and Transplantation, Karachi, 74200, Pakistan

## Abstract

The Kalash represent an enigmatic isolated population of Indo-European speakers who have been living for centuries in the Hindu Kush mountain ranges of present-day Pakistan. Previous Y chromosome and mitochondrial DNA markers provided no support for their claimed Greek descent following Alexander III of Macedon's invasion of this region, and analysis of autosomal loci provided evidence of a strong genetic bottleneck. To understand their origins and demography further, we genotyped 23 unrelated Kalash samples on the Illumina HumanOmni2.5M-8 BeadChip and sequenced one male individual at high coverage on an Illumina HiSeq 2000. Comparison with published data from ancient hunter-gatherers and European farmers showed that the Kalash share genetic drift with the Paleolithic Siberian hunter-gatherers and might represent an extremely drifted ancient northern Eurasian population that also contributed to European and Near Eastern ancestry. Since the split from other South Asian populations, the Kalash have maintained a low long-term effective population size (2,319–2,603) and experienced no detectable gene flow from their geographic neighbors in Pakistan or from other extant Eurasian populations. The mean time of divergence between the Kalash and other populations currently residing in this region was estimated to be 11,800 (95% confidence interval = 10,600−12,600) years ago, and thus they represent present-day descendants of some of the earliest migrants into the Indian sub-continent from West Asia.

## Introduction

Human populations show subtle allele-frequency differences that lead to geographical structure, and available methods thus allow individuals to be clustered according to genetic information into groups that correspond to geographical regions. In an early worldwide survey of this kind, division into five clusters unsurprisingly identified (1) Africans, (2) a widespread group including Europeans, Middle Easterners, and South Asians, (3) East Asians, (4) Oceanians, and (5) Native Americans. However, division into six groups led to a more surprising finding: the sixth group consisted of a single population, the Kalash.^1^ The Kalash are an isolated South Asian population of Indo-European speakers residing in the Hindu Kush mountain valleys in northwest Pakistan, near the Afghan frontier. With a reported census size of 5,000 individuals, they represent a religious minority with unique and rich cultural traditions. DNA samples from the Kalash have been distributed as part of the cell-line panel from the Foundation Jean Dausset’s Human Genome Diversity Project and Centre d’Etude du Polymorphisme Humain (HGDP-CEPH) for over a decade and have formed part of several genetic analyses.^2^ Analyses of uni-parental (Y chromosome and mitochondrial) DNA markers characterized the Kalash as a small population that had undergone a population bottleneck during their recent migration to their present-day abode.^3,4^ This was confirmed by the study of genome-wide autosomal SNPs, which highlighted a strong pattern of genetic drift in this population.^5^ A recent exploration of admixture at fine scales suggested that a major admixture event between the Kalash and present-day western Eurasians occurred between 990 and 210 BCE and related this to Alexander’s invasion of the Indian sub-continent in 327–326 BCE,^6^ although no evidence of such admixture was detected by an analysis of Y chromosome and autosomal short tandem repeat (STR) variation in the Kalash.^7,8^

To further investigate the Kalash population’s demographic history and origins, we genotyped additional unrelated Kalash samples on the Illumina bead chip and sequenced one male individual at high coverage. Our aim was to assess whether the Kalash were a recent or an ancient isolate and categorize the extent of genetic isolation and admixture, if any, with extant or archaic humans and thus better understand the reasons for their unique position in worldwide comparisons.

## Material and Methods

### DNA Samples and Genotyping

**Figure 1:**
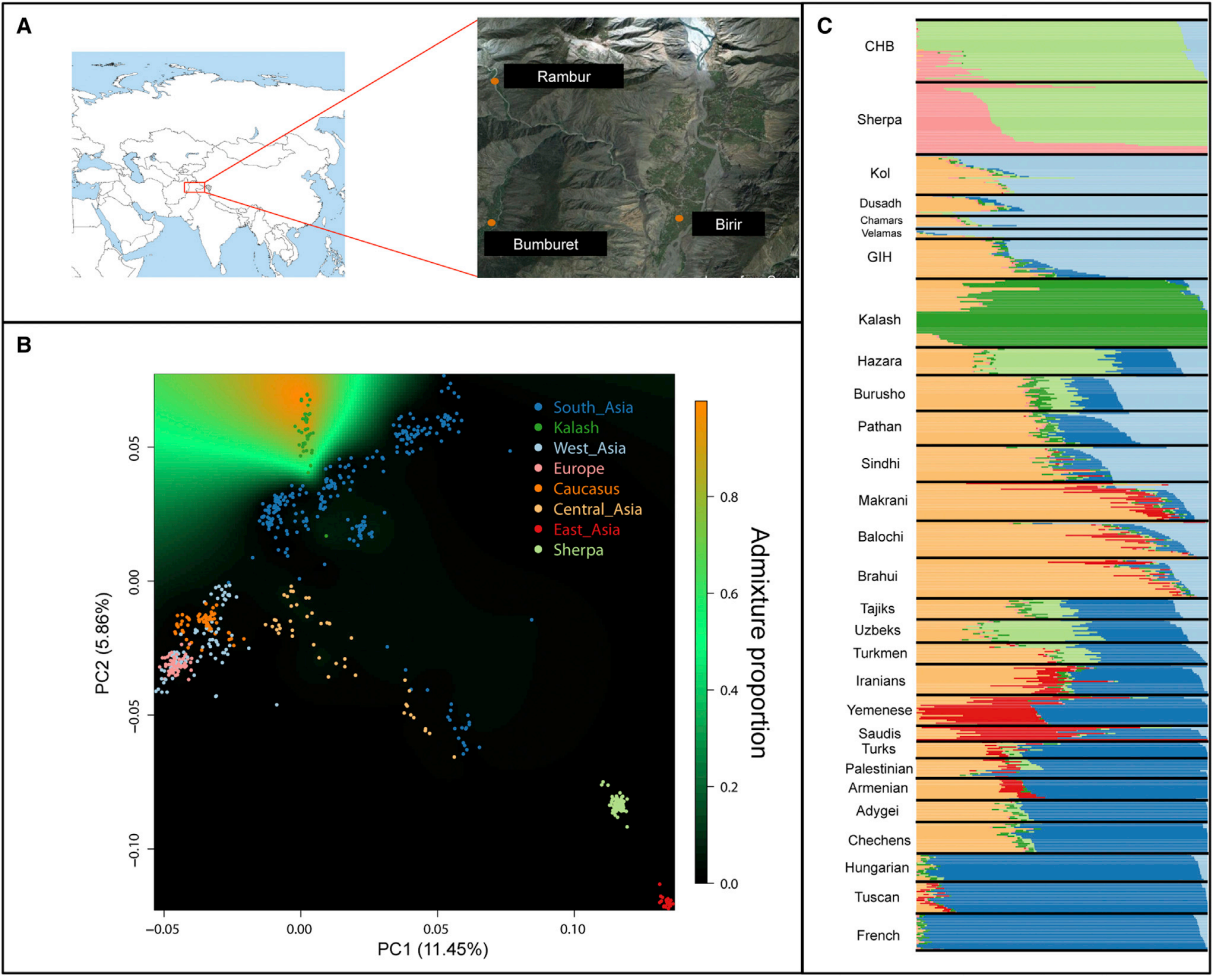
Population Structure and Isolation of the Kalash (A) Geographic location of the three Pakistani villages where the Kalash samples were collected. (B) Principal-component analysis (PCA) of Eurasian populations shows the first two components superimposed with the spatial kriging interpolation of the admixture coefficient of the Kalash genetic cluster. The proportion of admixture is indicated by color: orange represents the maximum level of admixture, and black represents the lowest. There is no gradient into the proportion of admixture with the Kalash cluster, suggesting a low level of gene flow between nearby populations and a high degree of isolation. (C) Admixture analysis in which the lowest cross-validation error (k = 7) shows the unique Kalash cluster (dark green).

The Kalash samples were collected from three valleys in the Hindu Kush mountain ranges in northwest Pakistan (Figure 1A). In accordance with the Declaration of Helsinki, the samples were collected after informed consent was obtained, and the study was approved by all relevant institutional ethics committees. Lymphoblastoid cell lines were established from all collected blood samples, and some (n = 25) were deposited with CEPH; these latter samples form part of the South and Central Asian collection of the HGDP-CEPH cell-line panel. We used 10 of these and an additional 13 samples that are not in the collection for our analysis. All of these unrelated (n = 23) Kalash males were genotyped on the Illumina HumanOmni2.5M-8 BeadChip with 200 ng of DNA (26 ng/μl) prepared from these lymphoblastoid cell lines.^2^ Genotyping calls and quality control (QC) were performed with GenoSNP^9^ by the Sanger Institute’s core genotyping facility. Genotypes were called only for samples passing Sequenom genetic fingerprinting and gender concordance. These were run through the standard QC pipeline. All 23 samples passed a call-rate threshold of 95% and were used in the downstream analysis. Genotyping quality was assessed by comparison of 178,072 SNPs that overlapped the Illumina 650,000 K SNP chip.^5^ Ten of the Kalash samples analyzed in this study had also been genotyped on this platform, and the sample genotype concordance was 99.999%. Comparative data were obtained from 35 populations representing Africa, Europe, Caucasus, and West, Central, East, and South Asia (Table S1).^5,10–12^

### DNA Sequencing

High-coverage (30×) 100-bp paired-end sequencing of one of the genotyped male samples was carried out on an Illumina HiSeq 2000 with 5 μg of lymphoblastoid cell line DNA, standard library preparation, and analysis pipelines developed for the 1000 Genomes Project.^13^ The sequenced reads were mapped to the human GRCh37 reference sequence (UCSC Human Genome Browser hg19) used by the project (human_g1k_v37.fasta.gz). Variant annotations were performed with the R package NCBI2R and Ensembl’s Variant Effect Predictor.^14^ There was high concordance (99.9%) between the variant calls from the high-coverage data and the same sample’s SNP-chip genotypes.

### Data Analysis

The data were merged with reference-population data from African and non-African sources covering Eurasia (Table S1).^5,10–12,15,16^ The merged dataset was pruned for the removal of variants in high (r^2^ > 0.4) linkage disequilibrium (LD) and individuals with high identity by descent (IBD) (PLINK IBD score > 0.6) from the analysis. The high IBD threshold was chosen to account for the increased inbreeding levels introduced by the strong genetic drift experienced by the Kalash population. Principal-component analysis (PCA) was performed with EIGENSOFT v.5.01, and ancestry analysis was performed with ADMIXTURE v.1.22.^17^ Spatial kriging was used to quantify the spatial genetic heterogeneity by interpolating ancestry values (obtained from ADMIXTURE analysis) for each cluster; the principal-component eigenvectors were used as coordinates for each individual.^18^ Genetic structure and gene flow between populations was investigated via three different approaches: ALDER,^19^ three-population (*f*3) statistics,^20^ and TreeMix.^21^

We applied pairwise sequentially Markovian coalescent (PSMC) analysis to draw inferences about the long-term effective population sizes and times of divergence from ten high-coverage genomes, including the Kalash.^22^ The nine other genomes were sequenced by Complete Genomics and included three unrelated African populations (Yoruba in Ibadan, Nigeria [YRI]; Luhya in Webuye, Kenya [LWK]; Maasi in Kinyawa, Kenya [MKK]) and six non-African genomes from East Asia (Han Chinese in Beijing, China [CHB]; Japanese in Tokyo, Japan [JPT]), Europe (Utah residents with Northern and Western European ancestry from the CEPH collection [CEU]; Toscani in Italy [TSI]), South Asia (Gujarati Indian in Houston, Texas [GIH]) and America (Mexican Ancestry in Los Angeles, California [MXL]). Phasing was carried out with SHAPEIT2^23^ and the 1000 Genomes Project reference panel.^22^ We also estimated effective population size and time of divergence between the Kalash and other populations by analyzing LD patterns in SNP-chip data with the NeON R package.^24^

We assessed the genetic relatedness of ancient genomes to modern populations by computing outgroup *f*3 statistics.^20^ In the absence of admixture with the outgroup, the expected value of *f*3 (outgroup; A, B) is a function of the shared genetic history of A and B. We used the YRI as an outgroup to non-African populations and computed *f*3 statistic (YRI; ancient, X) to investigate the shared history of an ancient genome and a set of 32 worldwide populations, including the Kalash (X), and *f*3 statistic (YRI; Kalash, Y) to investigate the Kalash and a set of 32 worldwide populations and ancient genomes (Y). Ancient genomes used in the analysis included the Mal’ta boy (MA-1), a Paleolithic Siberian hunter-gatherer;^25^ La Braña 1, a Mesolithic European hunter-gatherer from Iberia;^26^ and Ötzi, the Tyrolean Iceman and a representative European Neolithic farmer.^27^ BAM files of the ancient genomes were downloaded from the respective references and managed with Picard v.1.112, and the genotypes were called with the Genome Analysis Toolkit v.3.3.^28^ Data on DNA polymorphisms were stored in VCF files and managed with VCFtools.^29^

### Selection

Selection in the Kalash populations was estimated as described by Yi et al.^30^ In brief, we obtained estimates of the population time of divergence (T) from F_ST_ values and corrected for the effective population size of the population considered (N_ep_), as shown in

$$T=\frac{\log\left(1-F_{\mathrm{ST}}\right)}{\log\left(1-\frac{1}{2\times N_{\mathrm{ep}}}\right)}.$$

For each marker, we calculated T between the Kalash and CHB (T^KC^), between the Kalash and Balochi (T^KB^)—who were considered representative of the other Pakistani populations—and between the CHB and Balochi (T^CB^). The population branch statistic (PBS), which defines the length of the branch leading to the Kalash since the split from East Asians, is equal to

$$\mathrm{PBS}=\frac{T^{\mathrm{KC}}+T^{\mathrm{KB}}-T^{\mathrm{CB}}}{2}.$$

N_ep_ estimates were obtained from the linkage data (2,471 [95% confidence interval (CI) = 2,319–2,603]) with the NeON R package. The variants within the 99^th^ percentile of the distribution of our PBS values were annotated, and a list of genes associated with these variants was used for Ingenuity Pathway Analysis (IPA).

### Simulation

We modeled the effect of drift and selection on specific variants by using the simuPOP library.^31^ We used the effective population size estimated from this study in the simulations and obtained the initial allele frequency for each marker from the observed data. We recorded the allele frequency every 50 generations for 500 generations (which roughly corresponds to 12,500 or 14,000 years ago if we assume a generation time of 25 or 28 years, respectively). Each scenario was replicated 1,000 times.

## Results

### The Kalash Are a Genetic Isolate

PCA using only Eurasian and South Asian populations separated the populations from Europe, Caucasus, and West Asia from East Asians in the first component and from South and Central Asians in the second component; Central Asians lay closer to the Sherpa from Nepal and CHB from East Asia. The Kalash samples clustered together as an outlier population to the other South Asian samples from India and Pakistan (Figure 1B). The Kalash genetic isolation was also supported by the ADMIXTURE plot (Figure 1C), in which the lowest cross-validation error was achieved with seven ancestry components. In this analysis, the Kalash were characterized mainly by a unique genetic component (dark green), although many samples shared a proportion of their ancestry with their neighbors in Pakistan (light orange and light blue). This light-blue component was also shared among many diverse populations from West, Central, and South Asia.

The pattern of runs of homozygosity (Figures S1A and S1B) and LD decay (Figure S2) reveal the highest average level of homozygosity in the Kalash and most extensive LD, possibly reflecting a high level of isolation and low effective population size. The *f*3 test statistic (Table S3), ALDER (Table S4), and TreeMix analysis (Figure S3) also test for admixture, but they showed no evidence of gene flow into the Kalash and thus provide further support for their genetic isolation. The TreeMix^5^ analysis supports most strongly an un-rooted tree that has 11 migration edges and shows extreme genetic drift in the Kalash but no migration events affecting them (Figure S3).

### The Kalash Are an Ancient Genetic Isolate

**Figure 2:**
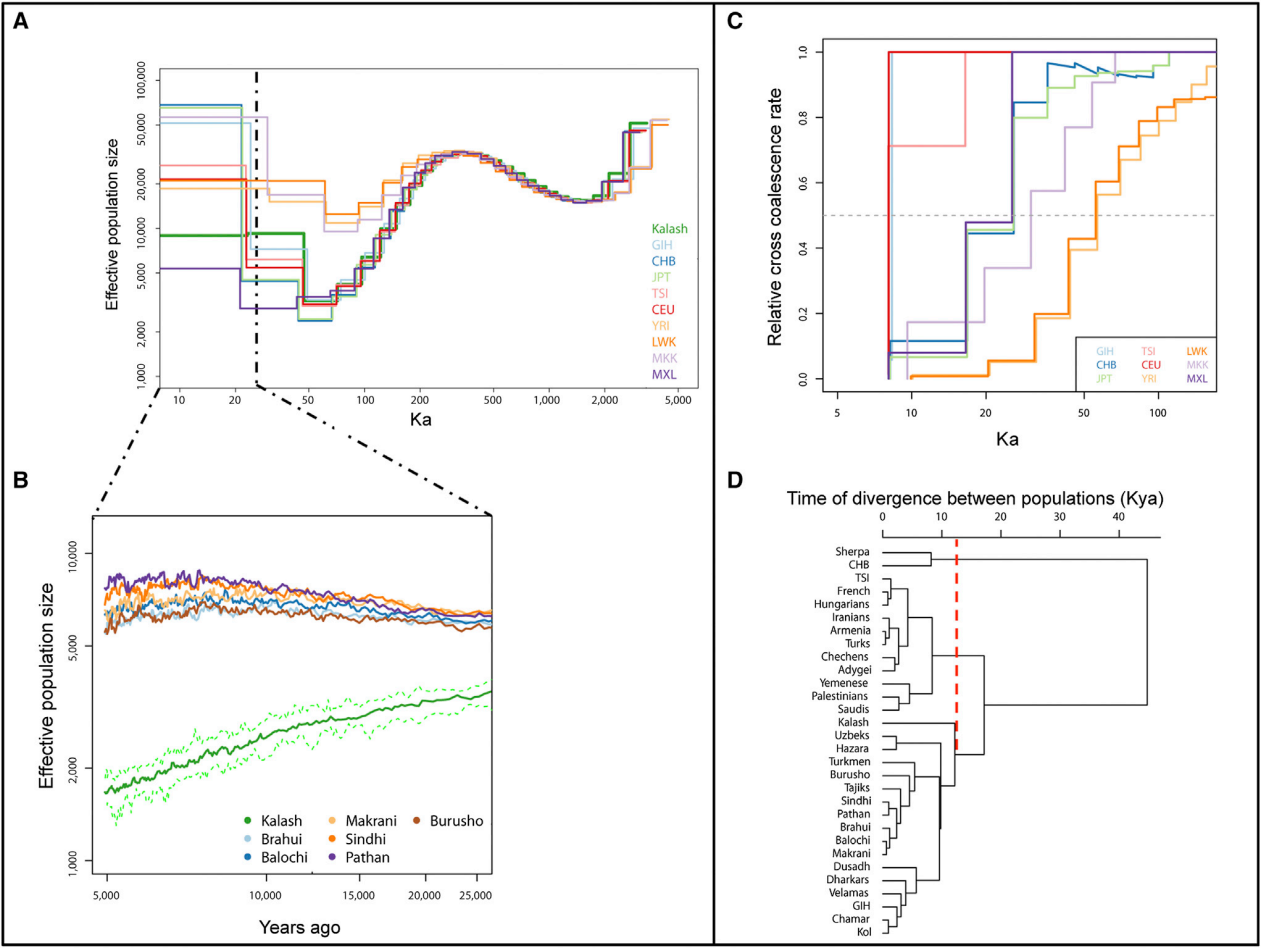
Kalash Demographic History (A) PSMC analysis shows a low effective population size for the Kalash. (B) Kalash effective population size estimated from LD analysis. (C) MSMC analysis of the time of the split between the Kalash and African genomes (YRI, LWK, and MKK) and non-African genomes from East Asia (CHB and JPT), Europe (CEU and TSI), South Asia (GIH), and America (MXL). (D) A UPGMA (unweighted pair group method with arithmetic mean) dendrogram shows the LD-estimated time of divergence between populations. The mean time of divergence between the Kalash and other populations from the Indian sub-continent is estimated to be 11,800 years ago (dashed red line).

PSMC analysis applied to the high-coverage Kalash, three African genomes (YRI, LWK, and MKK), and six non-African genomes showed that the Kalash, like other non-Africans, experienced a severe bottleneck 50,000–70,000 years ago. The Kalash recovered slightly after the bottleneck but never achieved an effective population size above 20,000, as observed in the GIH (the other South Asian genome) and other non-African genomes, except the MXL (Figure 2A). The Kalash have maintained a low effective size below 10,000 for more than 20,000 years before the present. This pattern of unusually small effective population size in the Kalash is also supported by the estimate from the decay of LD, which was significantly lower (p = < 2 × 10^−14^) than that of neighboring populations from Pakistan (Figure 2B), although the estimated absolute sizes differed between the two approaches.

To examine the time of divergence between the Kalash and other genomes, we used multiple sequentially Markovian coalescent (MSMC) analysis on phased high-coverage genomes. The estimates based on pairs of genomes showed that the Kalash split first from Africans (LWK, MKK, and YRI) and then from East Asians (CHB and JPT). The split from Europeans (CEU and TSI) and South Asians (GIH) appears to have happened around the same time (Figure 2C), approximately 8,000 years ago. Examination based on LD decay in genotyping data also showed that the Kalash were the first population to split from the Central and South Asian cluster around 11,800 (95% CI = 10,600–12,600) years ago (Figure 2D). This estimate was obtained by UPGMA (unweighted pair group method with arithmetic mean) phylogenetic analysis comparing the structure of the tree in the Kalash and other South and Central Asian populations. This split time remained constant even after the addition of the YRI population. We also estimated these split times by using different subsets of non-African populations. The resulting UPGMA trees were not strongly affected by different subsets of European or South Asian populations (Figure S5), and the split times between the Kalash and other populations ranged from 9,600 to 12,600 years ago.

### The Kalash Share Genetic Drift with Paleolithic Siberian Hunter-Gatherers

**Figure 3:**
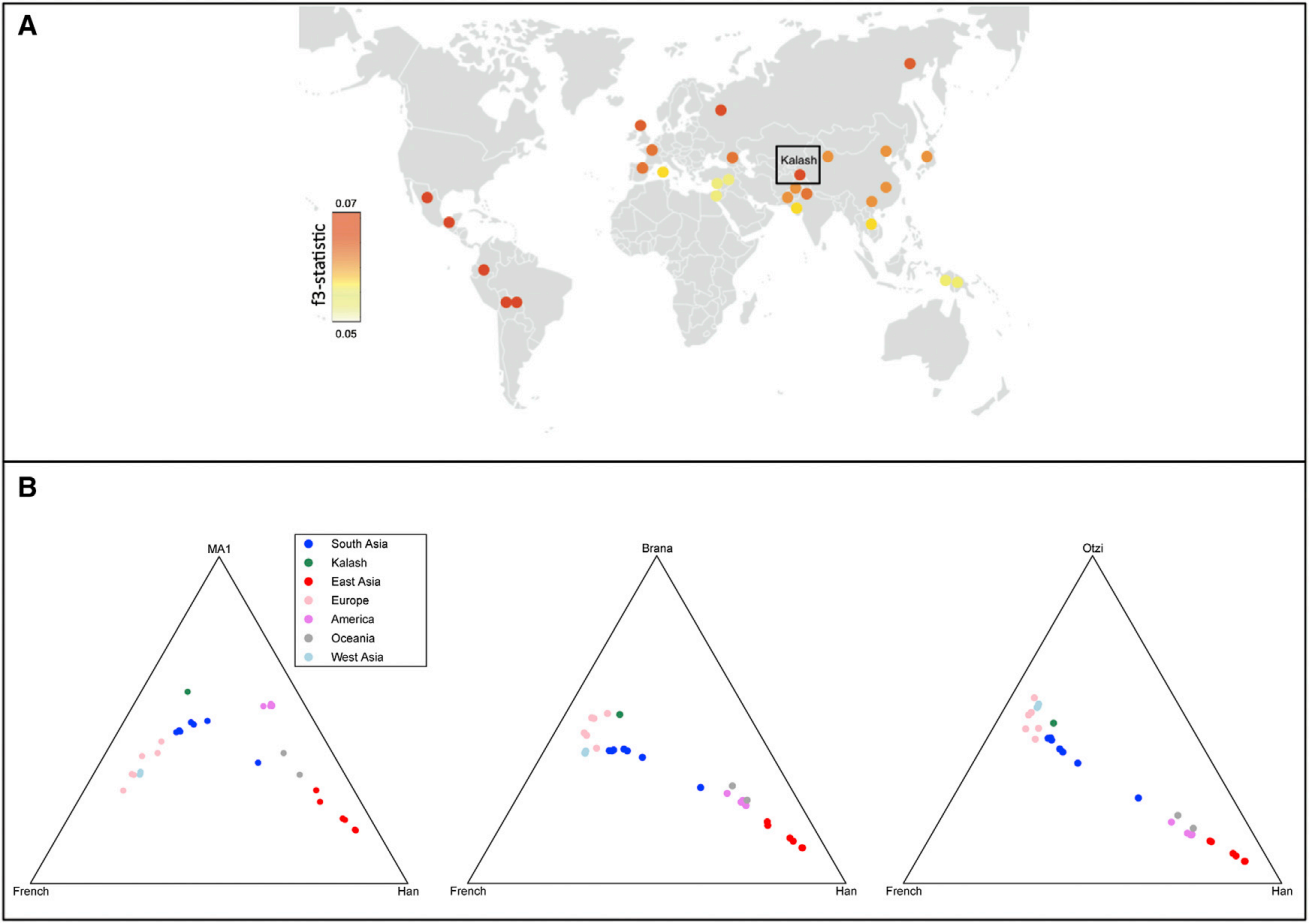
Shared Genetic Drift with Ancient Genomes (A) Proportion of shared genetic drift (measured as *f*3 statistics) between extant world-wide HGDP-CEPH populations (including the Kalash) and the ancient Siberian hunter-gatherer (MA-1). The magnitude of the computed *f*3 statistics is represented by the graded heat key. The proportion of genetic drift shared between the Kalash and MA-1 is comparable to that shared between MA-1 and the Yakut, Native Americans, and northern European populations. (B) Ternary plot of shared genetic drift with three ancient genomes: MA-1 (left), La Braña 1 (middle), and Ötzi, the Tyrolean Iceman (right). The high proportion of genetic drift shared between the Kalash and MA-1 is comparable to that shared between MA-1 and Native Americans. In comparison with other populations from South Asia, the Kalash also share a higher proportion of genetic drift with La Braña 1 and Ötzi.

We assessed the genetic relatedness between three ancient genomes and modern human populations, including the Kalash, by computing outgroup *f*3 statistics. The measure circumvents potential bias from classical genetic relatedness tests, such as PCA (which has sample-size bias) and F_ST_ (which is sensitive to genetic drift that has occurred since divergence of the test populations), when using ancient genomes. According to outgroup *f*3 statistics, the Kalash share a high level of genetic drift with MA-1, a Paleolithic Siberian hunter-gatherer skeleton dated to ∼24,000 years ago, but not a very high level with La Braña 1, the Mesolithic European hunter-gatherer (skeletal remains dated to ∼7,000 years ago) or the European farmers represented by Ötzi, the Tyrolean Iceman dated to ∼5,300 years ago (Figure 3). Similar to Native Americans, the Kalash share a high proportion of genetic drift with MA-1. In comparison with other populations from Pakistan and India, the Kalash also share a higher proportion of genetic drift with La Braña 1 and Ötzi. The level of drift shared with La Braña 1 and Ötzi is comparable to that of other North European populations (Figure 3B). We also used TreeMix to estimate the proportion of Neandertal ancestry from the high-coverage archaic Altai Neandertal who lived ∼50,000 years ago. The jackknife estimate of Neandertal-to-Kalash gene flow was 2.4% ± 0.48%.

### Consequences of Ancient Isolation

We also examined the effect of the inferred long-term isolation on genetic drift and natural selection in the Kalash. Our PBS analysis showed evidence of possible positive selection on 1,709 SNPs, of which 762 lie within 548 genes, including *RYR2* (MIM: 180902) and *ACTN3* (MIM: 102574) (Table S5). IPA showed an enrichment of selection signals in 28 genes associated with cardiovascular physiology and disease pathways (Fisher’s exact test p value = 4.61 × 10^−9^).

**Figure 4:**
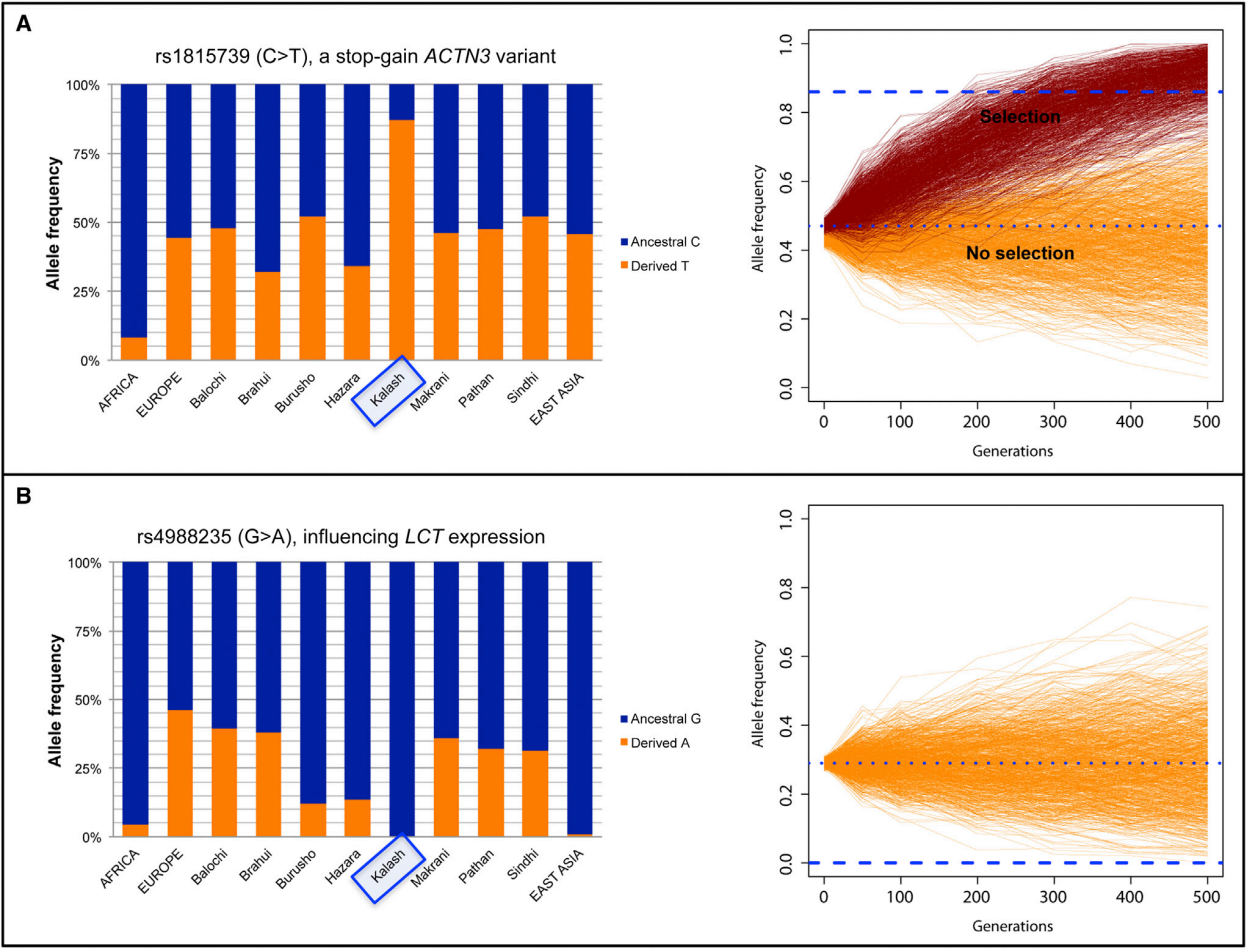
Consequences of Drift and Selection in the Kalash (A) A nonsense variant in *ACTN3* (rs1815739) is present at a higher frequency (left) in the Kalash than in their neighbors in Pakistan. Forward-time simulations (right) show that such a high frequency of the derived allele in the Kalash (dashed blue line) is only observed in a scenario that considers positive selection acting on the variant. The lower line represents the observed mean frequency of the derived allele in the Pakistani population, the orange lines represent the simulated allele frequency of the derived allele in each replicate in the scenario without selection, and the dark red lines represent each replicate in the scenario with positive selection. The observed frequency of the derived allele in Kalash population is reached only in the scenario with selection and only after 400 generations of drift (∼10,000 or 11,200 years ago if we assume a generation time of 25 or 28 years, respectively), suggesting that the observed pattern for this stop gain on *ACTN3* can best be explained by selection acting in ancient times and not by any recent population split. (B) The Kalash are fixed for the ancestral allele of the *MCM6* intronic variant (rs4988235) that is associated with lactose intolerance. The derived allele that is associated with lactase persistence is present at moderate frequency in populations from Pakistan (left panel and upper dashed line in the right panel). Forward-time simulations (right panel) suggest that recent isolation and genetic drift cannot explain the observed pattern for this functional polymorphism in the Kalash population. Only 1/1,000 replicates (represented by orange lines) reach fixation after 500 generations of drift (∼12,500 years ago if we assume a generation time of 25 years).

Two variants that were highly differentiated between the Kalash and the neighboring Pakistani populations stood out. One variant, rs4988235 (c.−13910C>T), which influences lactase (*LCT* [MIM: 603202]) expression and confers lactose tolerance, is fixed for the ancestral lactose-intolerant allele in the Kalash. The derived allele, however, is reported to be present at a moderate frequency (average 29%) in Pakistan.^32^ Forward-time simulations demonstrated that the observed pattern cannot easily be explained by recent genetic drift, given that only 0.1% of the 1,000 simulations achieved fixation for the ancestral allele after 500 generations (Figure 4A).

The second variant, rs1815739 (c.1729C>T [p.Arg577Ter]), is a natural knockout variant in *ACTN3* and has been associated with elite athletic performance.^33^ The derived T allele is present at a very high frequency (93%) in the Kalash. The average frequency in the remaining Pakistani populations is 47%. Using this (47%) frequency as a starting point for the forward-time simulations, we found that the very high frequency for this variant in the Kalash cannot be explained by genetic drift alone, even after 500 generations (Figure 4B). A selection signal (selection coefficient s = 0.01) achieved the observed frequency in 80% of the simulations after 500 generations. Both of these results support the long-standing isolation of the Kalash.

## Discussion

The present study sheds light on the origins of the enigmatic Kalash population from Pakistan. We propose that the population represents an ancient genetic isolate rather than a recently split population showing extreme genetic drift, as suggested by earlier studies.^1,6^ The outlier status of these South Asians is corroborated by the fact that we found no evidence of recent admixture in the Kalash by using a variety of analyses, including TreeMix, *f*3, and linkage-based statistics. The fact that researchers also genotyped ten of these samples earlier by using the HGDP-CEPH panel and that these cluster with the samples genotyped in this study rules out the possibility of confounding results due to population sub-structure within the Kalash.

The ancient separation of the Kalash from a common Eurasian ancestor is supported by PSMC and MSMC analyses, which estimated that the Kalash split from East Asians (CHB and JPT as proxy) prior to splitting from Europeans and other South Asian populations. The split from Europeans (CEU and TSI) and South Asians (represented here by GIH) appears to have occurred during the Neolithic period, which is also supported by the decay of LD. LD decay showed that the Kalash were the first population to split from the other Central and South Asian cluster around 11,800 (95% CI = 10,600−12,600) years ago. This estimate remained constant even after the addition of an African (YRI) population or when the Kalash were compared with different subsets of non-African populations. The pairwise times of divergence with other Pakistani populations ranged from 8,800 years ago with the Burusho to 12,200 years ago with the Hazara. Although migration and undetected admixture in reference populations could bias our estimate of the time of divergence, using different subsets of population revealed no strong bias in the split between the Kalash and South Asians, which occurred after the split between Europeans and South Asian populations.

Since this split, the Kalash have maintained a low N_e_ of around 2,500 (95% CI = 2,300–2,600), estimated from LD decay with no evidence of admixture. These N_e_ estimates are lower than those obtained from PSMC analysis because the latter method gives a single estimate of the cross-coalescence rate from the present to 24,000 years ago, whereas the linkage-based method gives us several estimates over the past 10,000 years. It is likely that PSMC analysis could not detect that the Kalash population suffered a continuous decline in effective population size. Taking into account the expected differences in N_e_ between autosomes and the Y chromosome, this is in agreement with the reported N_e_ of 237–1,124, which was estimated with observed and evolutionary mutation rates for Y chromosomal STRs.^34^

The Kalash represent a unique branch in the South Asian population tree and appear to be the earliest population to split from the ancestral Pakistani and Indian populations, indicating a complex scenario for population origins in the sub-continent rather than just the ancestral northern and southern Indian components identified previously.^35^ These Indo-European speakers were possibly the first migrants to arrive in the Indian sub-continent from northern or western Asia. This is supported by the higher level of shared genetic drift between the Kalash and the Paleolithic Siberian hunter-gatherer skeleton (MA-1) than between MA-1 and the other South Asian populations.

Whereas the Kalash have recently been reported to have European admixture, postulated to be related to Alexander’s invasion of South Asia,^6^ our results show no evidence of admixture. Although several oral traditions claim that the Kalash are descendants of Alexander’s soldiers, this was not supported by Y chromosomal analysis in which the Kalash had a high proportion of Y haplogroup L3a lineages, which are characterized by having the derived allele for the PK3 Y-SNP and are not found elsewhere.^7^ They also have predominantly western Eurasian mitochondrial lineages and no genetic affiliation with East Asians.^4^

We observed that the Kalash share a substantial proportion of drift with a Paleolithic ancient Siberian hunter-gatherer, who has been suggested to represent a third northern Eurasian genetic ancestry component for present-day Europeans.^36,37^ This is also supported by the shared drift observed between the Kalash and the Yamnaya, an ancient (2,000–1,800 BCE) Neolithic pastoralist culture that lived in the lower Volga and Don steppe lands of Russia and also shared ancestry with MA-1.^36,37^ Thus, the Kalash could be considered a genetically drifted ancient northern Eurasian population, and this shared ancient component was probably misattributed to recent admixture with western Europeans.

We also looked at how this long-term separation, isolation, and low effective population size affected the patterns of genetic variation in the Kalash. One striking example is the frequency of the derived allele for rs4988235, which has been linked to lactose tolerance. The Kalash, like the MA-1, are fixed for the ancestral allele for this variant, whereas their neighbors in Pakistan have been observed to have moderate frequencies of the derived allele. Although this supports their long-term isolation, it is surprising in other ways because the Kalash have no reported lactose intolerance and indeed celebrate a “milk day” during their annual spring rituals.^38^ This suggests that there might be additional derived lactase-persistence alleles in the *LCT*-*MCM6* (MIM: 601806) region in this population.

Another example is the extremely high frequency (93%) of the stop-gain *ACTN3* variant (rs1815739) associated with normal variation in human muscle strength and speed.^39^ This variant was picked up as an outlier in the PBS test for selection in the Kalash. Simulations indicated that such a high frequency of the derived allele in the Kalash can only be obtained under a scenario that includes positive selection. The variant might be relevant in cardiovascular conditioning and muscle strength related to climbing up and down high mountain passes. Although *ACTN3* has not been associated with adaptation to high altitude, *RYR2*, another gene with an intronic outlier variant (rs2992644) in PBS, has.^40^

It has been postulated that South Asia, which is now a densely occupied land, was encountered by the first populations of modern humans that ventured out of Africa more than 50,000 years ago. The exact route taken by these earliest settlers is not known, although it has been suggested that they traveled via a southern coastal route.^41,42^ The genetically isolated Kalash might be seen as descendants of the earliest migrants that took a route into Afghanistan and Pakistan and are most likely present-day genetically drifted representatives of these ancient northern Eurasians. A larger survey that includes populations from their ancestral homeland in Nuristan, Afghanistan, would provide more insights into their unique genetic structure and origins and help explain the complex history of the peopling of South Asia.

## Accession Numbers

The high-coverage Kalash sequence reported in this paper has been deposited in the European Nucleotide Archive (ENA) under accession number ENA: ERS233567.

## Web Resources

The URLs for data presented herein are as follows:

1000 Genomes, http://www.1000genomes.org
Ensembl, http://www.ensembl.org/index.html
European Nucleotide Archive, http://www.ebi.ac.uk/ena
HUGO Gene Nomenclature Committee, http://www.genenames.org/
Ingenuity Pathway Analysis, http://www.ingenuity.com
NCBI2R, http://cran.r-project.org/src/contrib/Archive/NCBI2R/
OMIM, http://omim.org
Picard 1.112, http://broadinstitute.github.io/picard/
UCSC Human Genome Browser, http://genome.ucsc.edu/cgi-bin/hgGateway
VCFtools, http://vcftools.sourceforge.net/

## Supplemental Data

Document S1. Figures S1–S5 and Tables S1 and S2

Table S3. *f*3 Test Results for the Kalash

Table S4. ALDER Results for the Kalash

Table S5. Results from Analysis of Population Branch Statistics

Document S2. Article plus Supplemental Data
