# Supplementary material for: The Kalash Genetic Isolate: Ancient Divergence, Drift, and Selection
Source: Am J Hum Genet. 2015 May 7;96(5):775–83. doi: 10.1016/j.ajhg.2015.03.012 (PMC4570283; doi:10.1016/j.ajhg.2015.03.012)
Supplement: Document S1. Figures S1–S5 and Tables S1 and S2 [file mmc1.pdf]

The American Journal of Human Genetics

Supplemental Data

The Kalash Genetic Isolate:

Ancient Divergence, Drift, and Selection

Qasim Ayub, Massimo Mezzavilla, Luca Pagani, Marc Haber, Aisha Mohyuddin, Shagufta Khaliq, Syed Qasim Mehdi, and Chris Tyler-Smith

Figure S1. Pattern of runs of homozygosity.

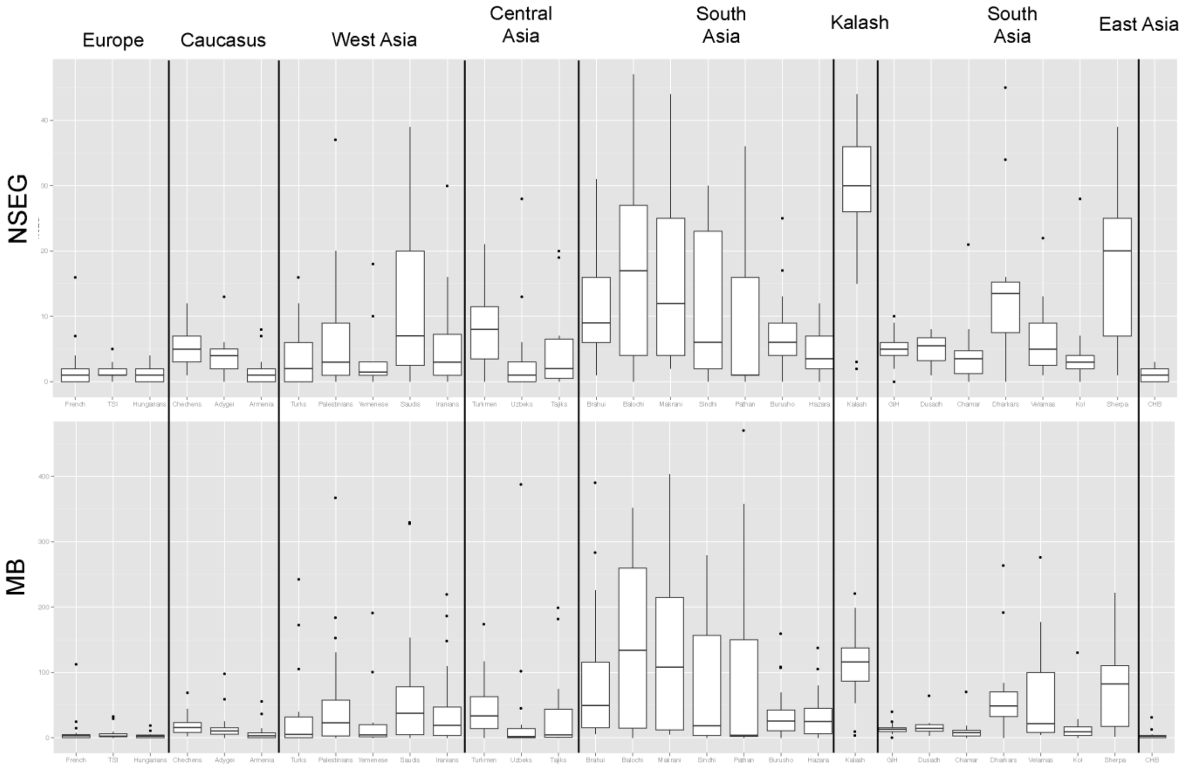

Number of homozygous segments (NSEG) and total level of homozygosity (MB) measured in Megabases.

Figure S2. Decay of linkage disequilibrium (LD) in the Kalash.

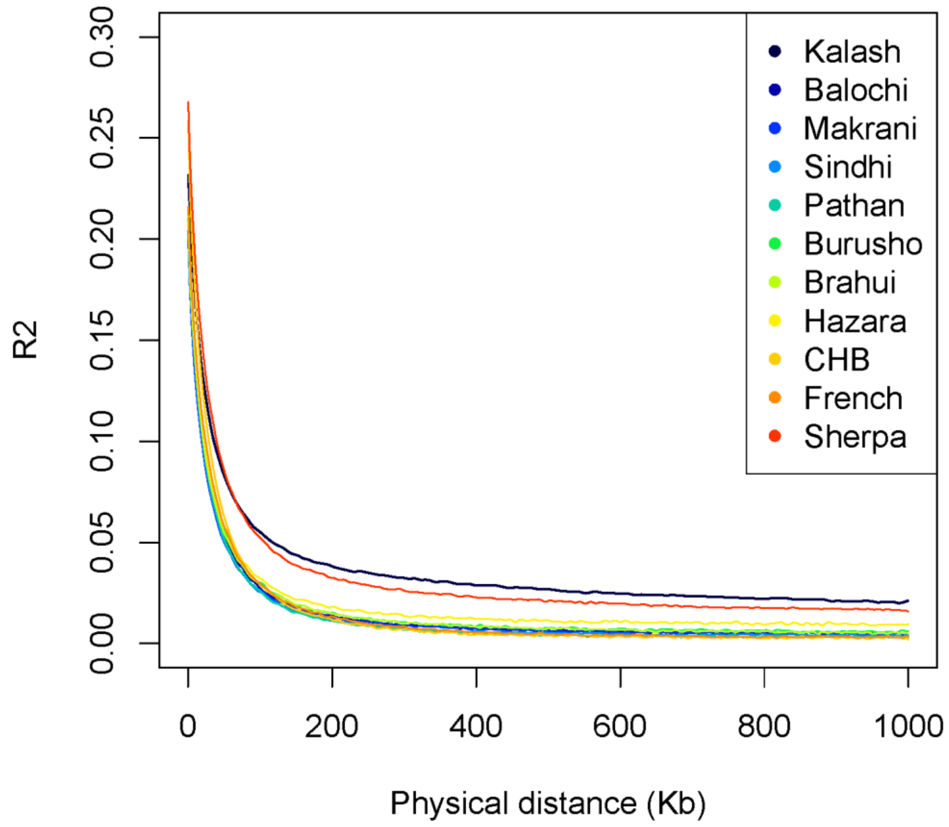

Kalash LD decay is comparable to the Sherpa and higher than any other Pakistani population.

Figure S3. TreeMix shows no gene flow in the Kalash.

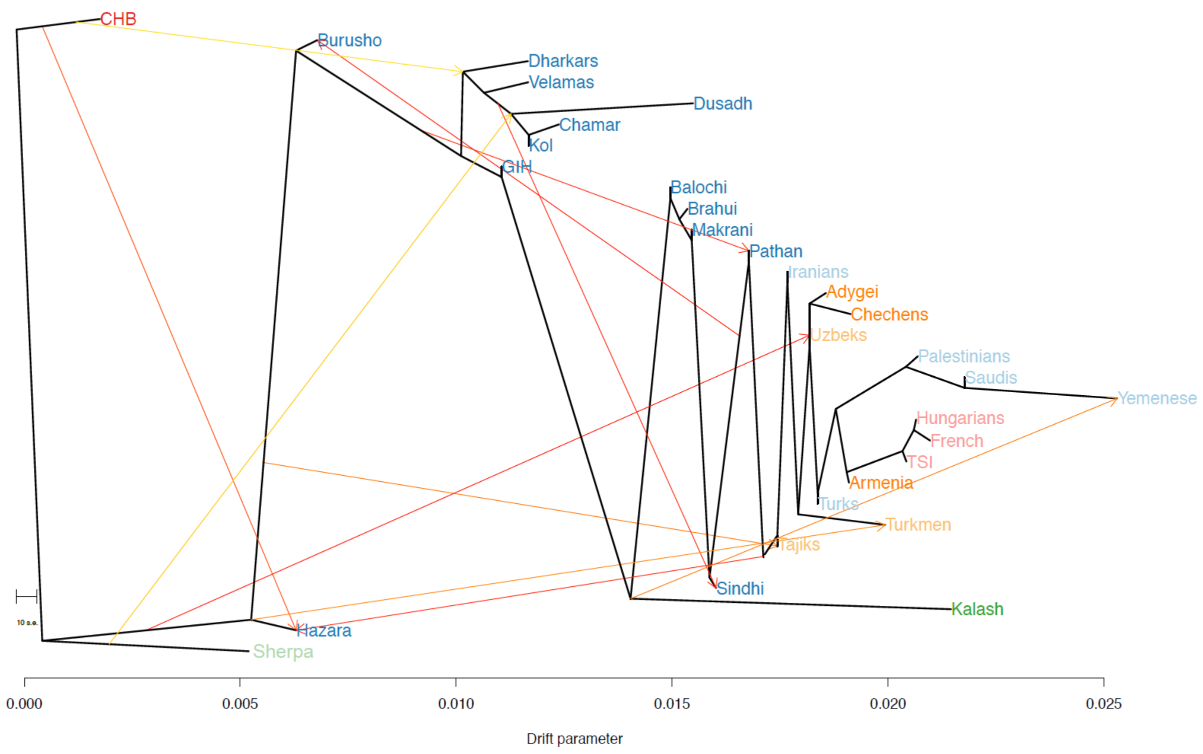

TreeMix analysis showing that the Kalash lie on a long branch among the other samples from Pakistan, suggestive of a high level of genetic drift with no evidence for gene flow.

Figure S4. Per-SNP population branch statistics.

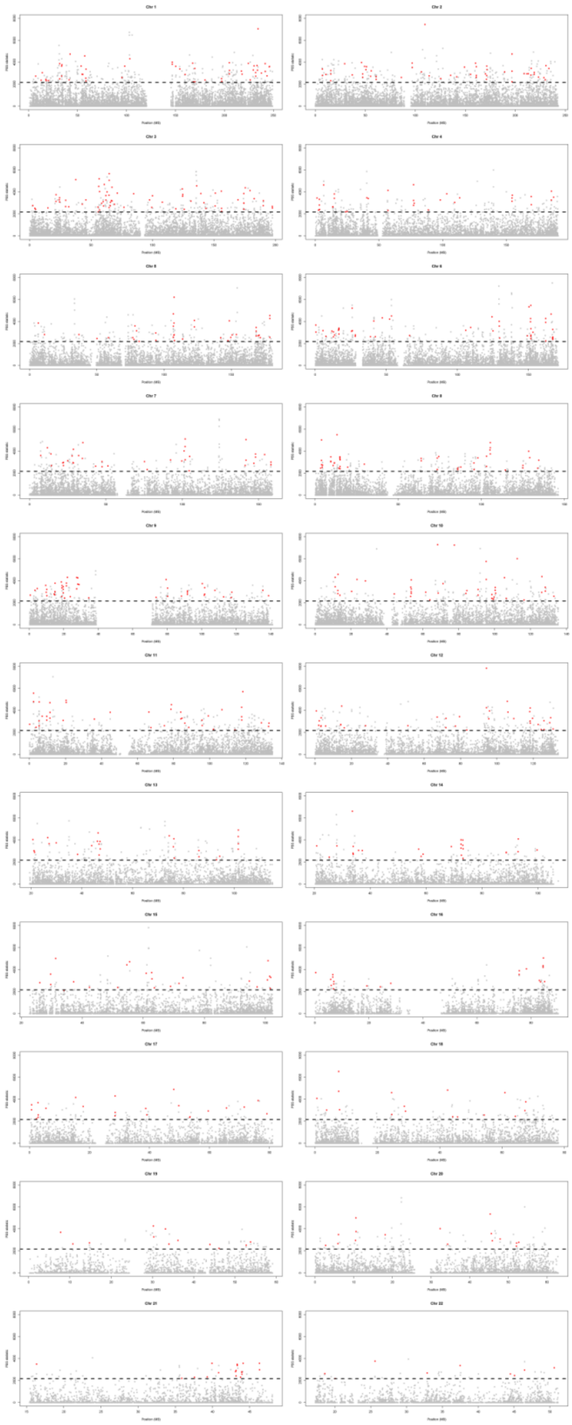

Results for the analyses of the Kalash population. Black dotted line refers to the 99th percentile of the distribution. Red points refer to the SNPs inside the genes reported in Table S5.

Figure S5. UPGMA tree using East Asian (CHB), Europeans (French, TSI) and South Asian (GIH) reference populations.

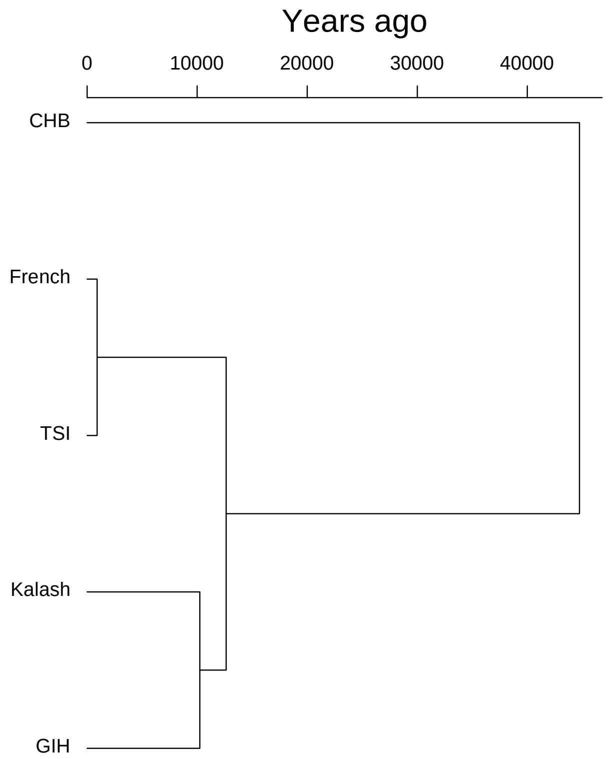

CHB are Han Chinese from Beijing, China, TSI are Tuscans in Italy and GIH are Gujarati Indian in Houston, Texas, a representative population from South Asia.

Table S1. Populations examined and their sample sizes.

Population	Sample Size	Region	References
Adygei	17	Caucasus	Behar et al., 2010[12]
Armenia	19	Caucasus	Behar et al., 2010[12]
Balochi	25	South Asia	Li et al., 2008[5]
Brahui	25	South Asia	Li et al., 2008[5]
Burusho	25	South Asia	Li et al., 2008[5]
Chamar	10	South Asia	Metspalu et al., 2011[15]
CHB	21	East Asia	The International HapMap 3 Consortium, 2010[16]
Chechens	20	Caucasus	Yunusbayev et al., 2012[11]
Dharkars	12	South Asia	Metspalu et al., 2011[15]
Dushadh	10	South Asia	Metspalu et al., 2011[15]
French	25	Europe	Li et al., 2008[5]
GIH	24	South Asia	The International HapMap 3 Consortium, 2010[16]
Hazara	20	South Asia	Li et al., 2008[5]
Hungarians	20	Europe	Behar et al., 2010[12]
Iranians	20	West Asia	Behar et al., 2010[12]
Kalash	23	South Asia	This study
Kalash	14	South Asia	Li et al., 2008[5]
Kol	17	South Asia	Metspalu et al., 2011[15]
Makrani	25	South Asia	Li et al., 2008[5]
Palestinians	25	West Asia	Li et al., 2008[5]
Pathan	23	South Asia	Li et al., 2008[5]
Saudi Arabian	20	West Asia	Behar et al., 2010[12]
Sherpa	68	South Asia	Jeong et al., 2014[10]
Sindhi	25	South Asia	Li et al., 2008[5]
Tajiks	15	Central Asia	Yunusbayev et al., 2012[11]
TSI	20	Europe	The International HapMap 3 Consortium, 2010[16]
Turkmen	15	Central Asia	Yunusbayev et al., 2012[11]
Turks	19	West Asia	Behar et al., 2010[12]
Uzbeks	15	Central Asia	Behar et al., 2010[12]
Velamas	10	South Asia	Metspalu et al., 2011[15]
Yemenese	9	West Asia	Behar et al., 2010[12]
Yoruba	21	Africa	Li et al., 2008[5]
San	5	Africa	Li et al., 2008[5]
Bantu	19	Africa	Li et al., 2008[5]
Biaka Pygmies	22	Africa	Li et al., 2008[5]
Mbuti Pygmies	13	Africa	Li et al., 2008[5]
Mandenka	22	Africa	Li et al., 2008[5]

Table S2. Long term effective population sizes estimated from linkage disequilibrium patterns.

Population	Long term Ne	95% CI
Adygei	6168	(5820-6570)
Armenia	6876	(6335-7373)
Balochi	6660	(6219-7414)
Brahui	6343	(5979-6898)
Burusho	6220	(5818-6958)
Chamar	5420	(5032-5899)
CHB	6918	(6360-7526)
Chechens	5990	(5345-6399)
Dharkars	4505	(3786-5020)
Dusadh	3031	(2690-3348)
French	6190	(5864-6722)
GIH	7369	(7070-7826)
Hazara	5823	(5395-6580)
Hungarians	6293	(5780-6911)
Iranians	7161	(6570-7746)
**Kalash**	**2471**	**(2319-2603)**
Kol	6809	(6013-7205)
Makrani	7022	(6510-7582)
Palestinians	6463	(5902-6856)
Pathan	7542	(6965-7948)
Saudis	6520	(5879-7007)
Sherpa	3394	(3184-3697)
Sindhi	7343	(7009-8094)
Tajiks	6738	(6396-7284)
TSI	6900	(6419-7367)
Turkmen	4713	(4223-5215)
Turks	7325	(6874-8119)
Uzbeks	6697	(5943-7305)
Velamas	4655	(4153-5342)
Yemenese	3409	(2951-3656)
Yoruba	10805	(10190-11200)
